# The Mechanism of Long Non-coding RNA in Cancer Radioresistance/Radiosensitivity: A Systematic Review

**DOI:** 10.3389/fphar.2022.879704

**Published:** 2022-05-05

**Authors:** Wenhan Wu, Shijian Zhang, Jia He

**Affiliations:** ^1^ Department of General Surgery (Gastrointestinal Surgery), The Affiliated Hospital of Southwest Medical University, Luzhou, China; ^2^ School of Clinical Medicine, Southwest Medical University, Luzhou, China; ^3^ Faculty Affairs and Human Resources Management Department, Southwest Medical University, Luzhou, China

**Keywords:** long non-coding RNA, cancer, radioresistance, radiosensitivity, systematic review

## Abstract

**Background and purpose:** Radioresistance remains a significant challenge in tumor therapy. This systematic review aims to demonstrate the role of long non-coding RNA (lncRNA) in cancer radioresistance/radiosensitivity.

**Material and methods:** The electronic databases Pubmed, Embase, and Google Scholar were searched from January 2000 to December 2021 to identify studies addressing the mechanisms of lncRNAs in tumor radioresistance/sensitivity, each of which required both *in vivo* and *in vitro* experiments.

**Results:** Among the 87 studies identified, lncRNAs were implicated in tumor radioresistance/sensitivity mainly in three paradigms. 1) lncRNAs act on microRNA (miRNA) by means of a sponge, and their downstream signals include some specific molecular biological processes (DNA repair and chromosome stabilization, mRNA or protein stabilization, cell cycle and proliferation, apoptosis-related pathways, autophagy-related pathways, epithelial-mesenchymal transition (EMT), cellular energy metabolism) and some signaling mediators (transcription factors, kinases, some important signal transduction pathways) that regulate various biological processes. 2) lncRNAs directly interact with proteins, affecting the cell cycle and autophagy to contribute to tumor radioresistance. 3) lncRNAs act like transcription factors to initiate downstream signaling pathways and participate in tumor radioresistance.

**Conclusion:** lncRNAs are important regulators involved in tumor radioresistance\sensitivity. Different lncRNAs may participate in the radioresistance with the same regulatory paradigm, and the same lncRNAs may also participate in the radioresistance in different ways. Future research should focus more on comprehensively characterizing the mechanisms of lncRNAs in tumor radioresistance to help us identify corresponding novel biomarkers and develop new lncRNA-based methods to improve radioresistance.

## Introduction

Radiotherapy is a standard treatment for many malignant tumors. About half of tumor patients receive this treatment, including radical, adjuvant, and palliative radiotherapy (Delaney et al., 2005; Schaue and McBride, 2015). Some tumors are sensitive to radiotherapy, which can achieve a radical cure. Some malignant tumors can be cured by a combination of radiotherapy, surgery, and chemotherapy. Besides, radiotherapy can also improve the quality of life and prolong the survival of patients with advanced tumors. The impact of radiotherapy on tumors is a complex process encompassing multiple factors and mechanisms. During radiotherapy, radiation can directly act on biologically active macromolecules, such as DNA and enzymes, causing abnormalities in their structure and function. It can also cause ionization and excitation of water molecules, producing free radicals and resulting in secondary damage to biological macromolecules (Nascimento and Bradshaw, 2016). In addition, radiation may have a secondary effect by affecting neurohumoral disorders, changing the permeability of cell membranes and blood vessel walls, and causing toxemia (Sharma et al., 2001; Wang et al., 2018).

While radiotherapy improves the prognosis of tumor patients, tumor cells exhibit varying degrees of resistance to it. The cancer radioresistance directly affects the effectiveness of radiation treatment on tumors, which is closely related to the poor prognosis in patients. Accordingly, tumor cells may possess mechanisms of radiotherapy resistance. However, the molecular mechanisms of radiation resistance of tumor cells remain poorly understood. Obviously, these mechanisms are complex and require more extensive characterization.

The long non-coding RNA (lncRNA) is a type of non-coding RNA with a length greater than 200 nucleotides. They participate in various cellular processes and are involved in the development of diseases (Mercer et al., 2009). It has been estimated that the human genome encodes more than 28,000 different lncRNAs (Tragante et al., 2014). Generally, the expression of lncRNA is typically lower than that of protein-coding genes and is highly tissue and time specific (Derrien et al., 2012). Evidence suggests that lncRNA can participate in multiple biological processes of tumor cells through various mechanisms, such as signal molecules, decoy molecules, guide molecules, scaffold molecules, and RNA sponges (Yang et al., 2014). More recent evidence indicates that lncRNA can modulate radiotherapy response by regulating key signal pathways, including DNA damage repair, cell apoptosis, cell metabolism, and autophagy (Podralska et al., 2020). However, to the best of our knowledge, no systematic review has been published to summarize the mechanisms of lncRNA in cancer radiotherapy resistance.

This study aimed to systematically review the literature and summarize the mechanism by which lncRNA contributes to cancer radioresistance/radiosensitivity. These findings may provide new insights for improving the efficiency of tumor radiotherapy, discovering new therapeutic targets, and translational medicine in the future.

## Material and Methods

This research strictly followed the PRISMA (Preferred Reporting Items for Systematic review and Meta-analyses) (Moher et al., 2009). This study was a systematic review and did not directly involve the issue of humans, so the review of the Institutional Review Board (IRB) was exempted.

### Search Strategy

The databases Pubmed, Embase, and Google Scholar were used for literature search. The search period for the literature was set from 1 January 2000 to 31 December 2021, and the language was restricted to English.

This study adopted the strategy of combining Pubmed mesh term and free words to determine the search terms, such as “neoplasms”, “cancer”, “tumor”, “RNA, long noncoding”, “long non-coding RNA, “lncRNA”, “radiotherapy”, “ionizing radiation”, “ionizing”, “radiation”, “radioresistance”, and “radiosensitivity”. The search strategy based on Pubmed was shown in Supplementary Table S1.

### Exclusion and Inclusion Criteria

Two researchers (Wenhan Wu and Shijian Zhang) independently searched and screened the literature using inclusion and exclusion criteria. We excluded irrelevant records based on the titles and abstracts and carefully evaluated the full text of the remaining documents. If there was a dispute between the two researchers, a third researcher (Jia He) would resolve the matter independently, and a consensus would be reached. Where possible, the original authors were contacted for more detailed data.

### Inclusion Criteria

1) Human tumor subjects; 2) the expression of lncRNA and cancer; 3) availability of data for both *in vitro* cell lines and animal studies; 4) lncRNAs involvement in cancer radioresistance/radiosensitivity and their specific mechanism.

### Exclusion Criteria

1) Only including vitro cell lines or animal studies; 2) review, editorial, and case reports; 3) incomplete data or uncertain mechanism.

### Data Extraction and Data Items

The literature included in this study has analyzed the relationship between lncRNA and cancer radioresistance/radiosensitivity in specific tumors, and determined their corresponding mechanism. We extracted the following data from each article: author, publication date, title, type of tumor and radiotherapy, involved lncRNAs and their expression levels, type of cell line, and corresponding mechanism of lncRNA.

## Results

### Study Search and Characteristics

**FIGURE 1:**
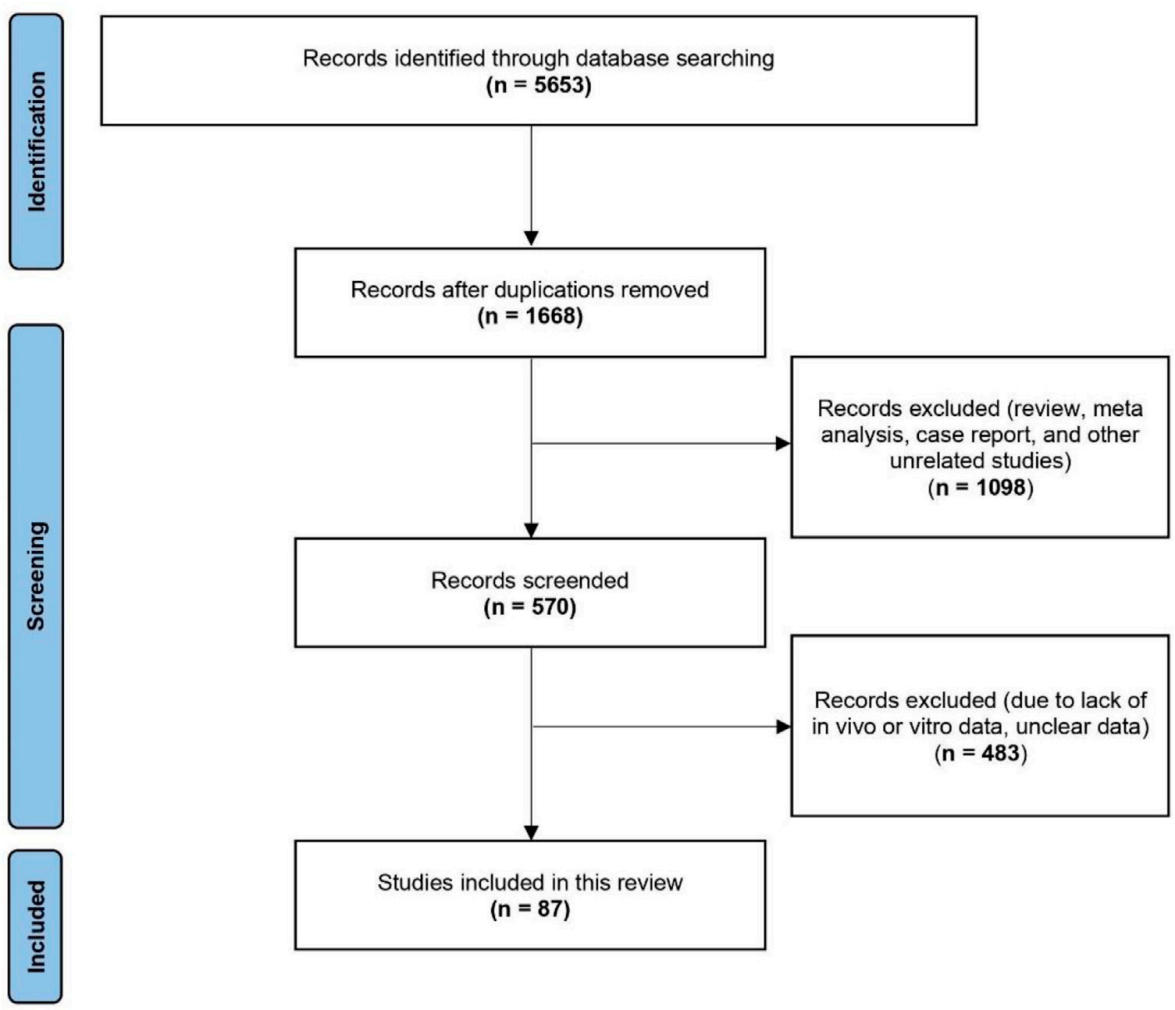
Flow chart of literature search and selection.

A diagram illustrating the literature search and selection process was shown in Figure 1. In the initial search, we identified a total of 5,653 potentially relevant documents. After removing duplicate publications, 1,668 studies remained. Based on the inclusion and exclusion criteria, we then carefully reviewed the abstracts of these studies and excluded 1,098 records, including reviews, meta-analyses, case reports, and other unrelated studies. We further evaluated the full text of the remaining 570 studies and excluded 483 studies. Because these excluded studies lacked *in vivo* or *in vitro* data, or their data was unclear. Finally, A total of 87 articles were included in this study.

Supplementary Table S2 listed the lncRNAs involved in cancer radioresistance/radiosensitivity and their concise mechanisms. The sources of cancer included bladder cancer (Tan et al., 2015; Jiang et al., 2017), breast cancer (Liu et al., 2019a; Zhang et al., 2019a; Wang et al., 2019; Qian et al., 2020; Zhang et al., 2020a), cardiac cancer (Jia et al., 2019), gastric cancer (Jiang et al., 2020; Lu et al., 2020; Xiao et al., 2020), cervical cancer (Jing et al., 2015; Li et al., 2018a; Han et al., 2018; Gao et al., 2019; Zhao et al., 2019; Wang et al., 2020a; Ge et al., 2020), colorectal cancer (Liu et al., 2020a; Liu et al., 2020b; Li et al., 2021; Liang et al., 2021), esophageal cancer (Li et al., 2017; Chen et al., 2018a; Zhang et al., 2019b; Wang et al., 2020b; Cheng et al., 2020; Liu et al., 2021a; Sun et al., 2021; Yang et al., 2021), glioblastoma (Li et al., 2018b; Ahmadov et al., 2021; Li et al., 2022), glioma (Yang et al., 2016; Zheng et al., 2016; Wang et al., 2020c; Wang et al., 2020d; Tang et al., 2020; Zheng et al., 2020; Gao et al., 2021; Tian et al., 2021), head and neck squamous cell cancer (Li et al., 2020a), laryngeal cancer (Cui et al., 2019; Tang et al., 2019; Tang and Shan, 2019), nasopharyngeal cancer (Jin et al., 2016; Wang et al., 2017; Ma et al., 2018; Yi et al., 2019; Han et al., 2020a; Han et al., 2020b; Wang et al., 2020e; Zhong et al., 2020; Wang et al., 2021a; Liu et al., 2021b; Guo et al., 2021), hepatocellular cancer (Chen et al., 2018b; Song et al., 2019; Yang et al., 2020a; Jin et al., 2021; Yu et al., 2021), lung cancer (Chen et al., 2015; Wu et al., 2017; Xue et al., 2017; Liu et al., 2019b; Wang and Hu, 2019; Yang et al., 2019; Brownmiller et al., 2020; Han et al., 2020c; He et al., 2020; Hou et al., 2020; Yu et al., 2020; Wang et al., 2021b; Liu et al., 2021c; Jiang et al., 2021; Zhang et al., 2021), medulloblastoma (Zhu et al., 2021), melanoma (Cui et al., 2021; Liu et al., 2021d), neuroblastoma (Yang et al., 2020b; Mou et al., 2021), prostate cancer (Chen et al., 2018c; Ma et al., 2020; Xiu et al., 2020), renal cell cancer (Zhou et al., 2021), and thyroid cancer (Li et al., 2020b; Chen et al., 2021). Out of the 87 studies, a total of 11 types of lncRNA have been independently reported in at least two articles to participate in cancer radioresistance/radiosensitivity, including lncRNA HOTAIR (*n* = 7), lncRNA GAS5 (*n* = 5), lncRNA PVT1 (*n* = 4), lncRNA TUG1 (*n* = 4), lncRNA NEAT1 (*n* = 3), lncRNA DGCR5 (*n* = 2), lncRNA FAM201A (n = 2), lncRNA KCNQ1OT1 (*n* = 2), lncRNA LINC00958 (*n* = 2), lncRNA MALAT1 (*n* = 2), and lncRNA XIST (*n* = 2).

### Mechanism of lncRNA Contributing to Cancer Radioresistance/Radiosensitivity

#### Acting on miRNA by Acting as a Sponge to Regulate Downstream Signals

**FIGURE 2:**
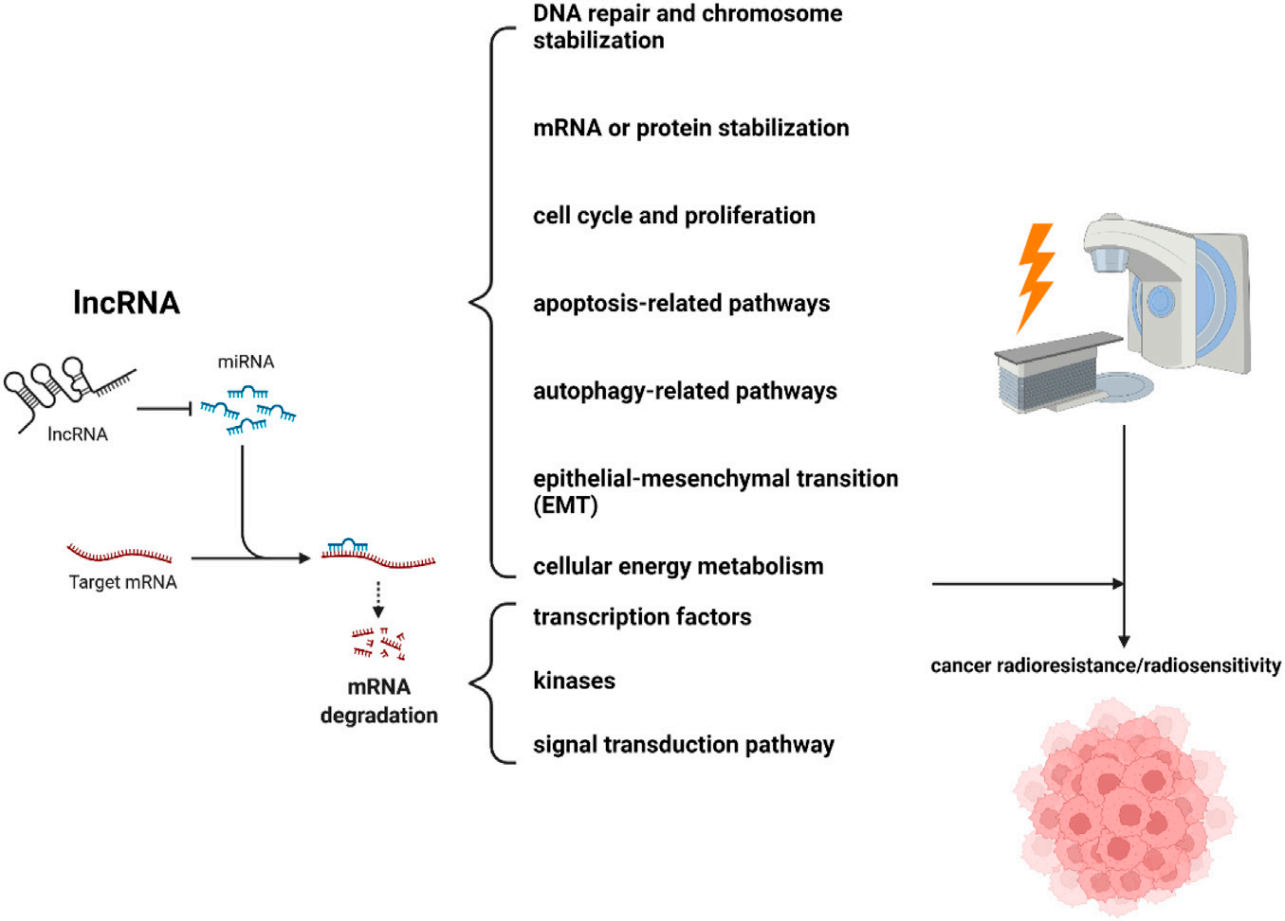
The mechanism of lncRNAs regulating ionizing radiation *via* miRNA.

Among the lncRNAs identified in this study, the majority acted as competitive endogenous RNA (ceRNA) in regulating cancer radioresistance/radiosensitivity. The fundamental mechanism was that lncRNA acted on microRNA (miRNA) by acting as a sponge to regulate downstream signals. In our study, the downstream signals that were regulated by the lncRNA/miRNA paradigm and mediated cancer radioresistance/radiosensitivity mainly included some specific molecular biological processes and some signaling mediators that regulate various biological processes (Figure 2). These specific molecular biological processes mainly included DNA repair and chromosome stabilization, mRNA or protein stabilization, cell cycle and proliferation, apoptosis-related pathways, autophagy-related pathways, epithelial-mesenchymal transition (EMT), and cellular energy metabolism. Those signaling mediators primarily included transcription factors, kinases, or some important signal transduction pathways, which often promoted tumor radioresistance\sensitivity through various mechanisms. It is worth noting that a total of six lncRNA\miRNA downstream mechanisms were not fully elucidated, representing the direction of further research (Wu et al., 2017; Xue et al., 2017; Jia et al., 2019; Tang et al., 2019; Jiang et al., 2020; Jin et al., 2021).

##### Molecular Biological Process 1: DNA Repair and Chromosome Stabilization

DNA double-strand break (DSB) is the most common cellular damage induced by ionizing radiation. If it is not repaired correctly, it may cause chromosomal abnormalities and even cell death. There is growing evidence of the role of lncRNA in DNA repair and chromosome stabilization *via* miRNA. In gastric cancer, lncRNA LINC01436 was reported to upregulate radioresistance through miR-513a-5p/APE1 axis (Lu et al., 2020). APE1 (DNA-(apurinic or apyrimidinic site) endonuclease) is a protein with multiple functions. It usually participates in DNA damage repair through the DNA base excision repair (BER) pathway (Wierstra, 2013). There was evidence that the lncRNA LINC00958 enhanced radioresistance *via* miR-5095/RRM2 in cervical cancer (Zhao et al., 2019). RRM2 (ribonucleotide reductase regulatory subunit M2) catalyzes the conversion of ribonucleotides to deoxyribonucleotides, which is the rate-limiting enzyme for DNA synthesis or repair, and plays a crucial role in tumor cell DNA synthesis and proliferation (Zhong et al., 2016). In colorectal cancer, lncRNA lnc-RI enhanced radioresistance through miR-4727-5p/LIG4 (Liu et al., 2020b). LIG4 (DNA ligase 4) is a DNA ligase, which is essential for V(D)J recombination and DNA double-strand break (DSB) repair through non-homologous end joining (NHEJ) (Gu et al., 2007). In esophageal squamous cell cancer, lncRNA FAM201A was reported to upregulate radioresistance *via* miR-101/ATM axis (Chen et al., 2018a). ATM (ATM serine/threonine kinase) is an essential component of the response to DNA damage and the maintenance genome stability, which is the main repair protein involved in double-strand break (DSB) homologous recombination repair (HRR) induced by ionizing radiation (Qin et al., 2019). Besides, lncRNA NORAD was reported to upregulate esophageal squamous cell cancer radioresistance *via* miR-199-a1/EEPD1 (Sun et al., 2021). EEPD1 (endonuclease/exonuclease/phosphatase family domain containing 1) participates in DNA repair and maintains genome stability by promoting end excision and homologous recombination repair (Wu et al., 2015). In hepatocellular cancer, lncRNA ROR upregulated radioresistance *via* miR-145/RAD18 axis (Chen et al., 2018b). RAD18 (RAD18 E3 ubiquitin-protein ligase) participates in the post-replication repair of UV-damaged DNA. It plays a role in the duplication of damaged DNA in filling the gaps of the daughter strands (Cotta-Ramusino et al., 2011). In prostate cancer, lncRNA TUG1 enhanced radioresistance through miR-139-5p/SMC1A axis (Xiu et al., 2020). SMC1A (structural maintenance of chromosomes 1A) is an important part of the functional kinetochore, which helps correctly separate chromosomes during cell division. It is also considered to have potential DNA repair functions (Yazdi et al., 2002). In renal cell cancer, lncRNA LINC02532 upregulated radioresistance *via* miR-654-5p/YY1 axis (Zhou et al., 2021). YY1 (transcriptional repressor protein YY1) is a multifunctional transcription factor and a core component of the chromatin remodeling INO80 complex. It is involved in transcription regulation, DNA replication, and DNA repair (Wu et al., 2007).

##### Molecular Biological Process 2: mRNA or Protein Stabilization

During radiation-induced cellular stress, maintaining mRNA and protein stability helps minimize cell damage and facilitate cell survival. A study reported that the lncRNA HOTAIR enhanced radioresistance in breast cancer through the miR-449b-5p/HSPA1A axis (Zhang et al., 2020a). HSPA1A (heat shock protein family A member 1A) can stabilize existing proteins and mediate the correct folding of proteins in the cytoplasm and organelles, thereby protecting tumor cells and enhancing their recovery (Santos et al., 2017). lncRNA LINC00511 also upregulated breast cancer radioresistance *via* miR-185/STXBP4 axis (Liu et al., 2019a). STXBP4 (syntaxin binding protein 4) plays a role in translocating vesicles from the cytoplasm to the cell membrane, which has been shown to contribute to protein stability (Li et al., 2009). In non-small cell lung cancer, lncRNA PVT1 induced radioresistance through miR-424-5p/CARM1 (Wang and Hu, 2019), while CARM1 (coactivator associated arginine methyltransferase 1) is involved in DNA packaging, transcription regulation, pre-mRNA splicing, and mRNA stability (Yang and Bedford, 2013). Moreover, the lncRNA GAS5 was shown to enhance radiosensitivity through miR-362-5p/SMG1 axis in thyroid cancer (Li et al., 2020b). SMG1 (serine/threonine-protein kinase SMG1) is involved in both mRNA surveillance and genotoxic stress response pathways. Its consumption leads to spontaneous DNA damage and an increasing in sensitivity to ionizing radiation (IR) (Yamashita et al., 2001).

##### Molecular Biological Process 3: Cell Cycle and Proliferation

The dysfunctional regulation of the cell cycle and proliferation is also associated with cancer radioresistance/radiosensitivity. A study reported that lncRNA SNHG12 enhanced radioresistance in cervical cancer cells *via* miR-148a/CDK1 (Wang et al., 2020a). CDK1 (cyclin-dependent kinase 1) promotes the transition from the G2 to M phase of the cell cycle, thereby promoting the proliferation of tumor cells (Hirai et al., 1995). lncRNA NEAT1 was also reported to enhance cervical cancer radioresistance *via* miR-193b-3p/CCND1 axis (Han et al., 2018). CCND1 (cyclin D1) is a highly conserved cyclin, which is mainly involved in the transition of the G1/S phase of the cell cycle by regulating CDK (Jares et al., 2007). In glioblastoma, lncRNA RBPMS-AS1 downregulate radioresistance *via* miR-301a-3p/CAMTA1 axis (Li et al., 2022). CAMTA1 (calmodulin-binding transcription activator 1) is a transcription factor, which induces the expression of natriuretic peptide A (NPPA), an anti-proliferative cardiac hormone (Schraivogel et al., 2011). Besides, in nasopharyngeal cancer, lncRNA PTPRG-AS1 enhanced radioresistance *via* miR-194-3p/PRC1 (Yi et al., 2019). PRC1 (protein regulator of cytokinesis 1) is a protein involved in cytokinesis. This protein is present at high levels during the S and G2/M phases of mitosis, but when the cell exits mitosis and enters the G1 phase, its level drops sharply (Zhan et al., 2017a).

##### Molecular Biological Process 4: Apoptosis-Related Pathways

Radiation-induced DNA damage may activate apoptosis-related signaling pathways, and the anti-apoptotic mechanisms of tumor cells are directly involved in radioresistance. It has been reported that lncRNA GAS5 decreased radioresistance through miR-106b/IER3 in cervical cancer (Gao et al., 2019). IER3 (immediate early response 3) is controlled by many stimuli and cellular conditions. It plays a dual role in tumor cell growth control and apoptosis, depending on the cell type and related conditions (Jin et al., 2015). In glioma, lncRNA NCK1-AS1 upregulated radioresistance *via* miR-22-3p/IGF1R axis (Wang et al., 2020c). IGF1R (insulin-like growth factor 1 receptor) binds insulin-like growth factors with high affinity. It has tyrosine kinase activity. It is highly overexpressed in many malignant tissues and acts as an anti-apoptotic agent (Yuan et al., 2018). In laryngeal cancer, lncRNA HOTAIR enhanced radioresistance *via* miR-454-3p/E2F2 axis (Cui et al., 2019). E2F2 (E2F transcription factor 2) is a member of the E2F transcription factor family and plays an inhibitory role in p53-independent apoptosis induced by ionizing radiation (IR) (Wichmann et al., 2010). In non-small cell lung cancer, lncRNA CYTOR upregulated radioresistance *via* miR-206/PTMA axis (Jiang et al., 2021), and PTMA (prothymosin alpha) involved in inhibiting apoptosis (Malicet et al., 2006).

##### Molecular Biological Process 5: Autophagy-Related Pathways

The autophagy pathway mediates the degradation of dysfunctional organelles and promotes protein turnover, thereby promoting radioresistance as a means of survival and adaptation in the presence of ionizing radiation. In colorectal cancer, lncRNA HOTAIR enhanced radioresistance *via* miR-93/ATG12 axis (Liu et al., 2020a). ATG12 (autophagy-related 12) is mainly involved in the formation of autophagic vesicles, and plays a vital role in tumor maintenance and treatment resistance (Yun and Lee, 2018). In lung adenocarcinoma, lncRNA KCNQ1OT1 was also reported to upregulated radioresistance *via* miR-372-3p/ATG5 and ATG12 axis (He et al., 2020).

##### Molecular Biological Process 6: Epithelial-Mesenchymal Transition

EMT is the process that epithelial cells transform into mesenchymal cells and acquire the ability to migrate. The acquisition of EMT in tumor cells is associated with radioresistance and poor prognosis. lncRNA TUG1 has been found to enhance radioresistance in bladder cancer *via* miR-145/ZEB2 axis (Tan et al., 2015). ZEB2 (zinc finger E-box binding homeobox 2) usually functions as a repressor of DNA transcription in the nucleus and plays a crucial role in the EMT of tumor cells (Fardi et al., 2019). In nasopharyngeal cancer, lncRNA MINCR upregulated radioresistance *via* miR-223/ZEB1 (Zhong et al., 2020), and ZEB1 (zinc finger E-box binding homeobox 1) drives the induction of EMT by activating stem cell characteristics, immune evasion, and epigenetic reprogramming (Zhang et al., 2019c). In neuroblastoma, lncRNA XIST was reported to enhance radioresistance through miR-375/L1CAM axis (Yang et al., 2020b). L1CAM (L1 cell adhesion molecule) is a glycoprotein involved in cancer development, which plays a role in EMT primarily through interactions with other cell adhesion molecules, integrins, or growth factor receptors (Maten et al., 2019).

##### Molecular Biological Process 7: Cellular Energy Metabolism

Changes in the metabolic pathways of tumor cells are considered to be a hallmark of tumors, and these changes can lead to radioresistance. In neuroblastoma, lncRNA LINC01410 was found to upregulate radioresistance *via* miR-545-3p/HK2 axis (Mou et al., 2021). HK2 (hexokinase 2) is located in the outer membrane of mitochondria, participates in most glucose metabolism pathways, and is thought to be involved in the supply of tumor cells (Liu et al., 2019c). In melanoma, lncRNA LINC01224 was reported to upregulate radioresistance *via* miR-193a-5p/NR1D2 axis (Cui et al., 2021). NR1D2 (nuclear receptor subfamily 1 group D member 2) acts as a transcription inhibitor and may affect cancer carbohydrate and lipid metabolism (Yu et al., 2018).

##### Signaling Mediator 1: Transcription Factor

Besides, the downstream targets of lncRNAs mediated by miRNA and involved in tumor radioresistance/radiosensitivity also included some transcription factors, kinases, or some important signal transduction pathways. These regulated downstream targets often have multiple biological functions in tumor development. In gastric cancer, lncRNA TRPM2-AS enhanced radioresistance *via* miR-612/FOXM1 axis (Xiao et al., 2020). FOXM1 (Forkhead box protein M1) is a transcription factor that controls the cell cycle and is involved in repairing DNA breaks. FOXM1 stimulates cell proliferation by promoting cells to enter the S and M phases. It also contributes to angiogenesis, invasion, metastasis, and EMT in tumors (Wierstra, 2013). In head and neck squamous cell cancer, lncRNA LINC00520 enhanced radioresistance *via* miR-195/HOXA10 (Li et al., 2020a), while In lung cancer, lncRNA LINC00483 upregulated radioresistance *via* miR-144/HOXA10 (Yang et al., 2019). Also, in lung cancer, lncRNA LINC00461 enhanced radioresistance *via* miR-195/HOXA10 (Hou et al., 2020). HOXA10 (homeobox A10) is a DNA-binding transcription factor that may regulate fertility, embryo vitality, and hematopoietic lineage commitment. It is thought to be related to tumor cell proliferation, migration, and invasion (Carrera et al., 2015).

##### Signaling Mediator 2: Kinase

In breast cancer, lncRNA LINC00963 upregulated radioresistance *via* miR-324-3p/ACK1 axis (Zhang et al., 2019a). ACK1 (activated CDC42 kinase 1) is a serine/threonine-protein kinase that contributes to cancer migration, survival, and proliferation *via* regulating WWOX and AKT1 (Mahajan and Mahajan, 2013). In rectal cancer, lncRNA EGOT enhanced radioresistance *via* miR-211-5p/ErbB4 axis (Li et al., 2021). ErbB4 (erb-b2 receptor tyrosine kinase 4) is a single-pass type I membrane protein with multiple cysteine rich domains, a transmembrane domain, and a tyrosine kinase domain. It is related to cell proliferation and differentiation in tumors (Segers et al., 2020). In colorectal cancer, lncRNA LINC00958 upregulated radioresistance *via* miR-422a/MAPK1 axis (Liang et al., 2021). While in glioma, lncRNA TPTEP1 downregulated radioresistance through miR-106a-5p/P38 MAPK (Tang et al., 2020). Moreover, in nasopharyngeal cancer, lncRNA LINC00114 enhanced radioresistance *via* miR-203/ERK/JNK signaling pathway (Han et al., 2020a). There are three well-defined MAPK subfamilies in mammals: extracellular signal-regulated kinase (ERK), c-Jun N-terminal kinase (JNK), and p38 kinase. The activation of each MAPK signal follows a three-layer kinase module, in which MAP3K phosphorylates and activates MAP2K, and MAP2K phosphorylates and activates MAPK. Once activated, MAPK controls a variety of cellular responses, such as tumor proliferation, differentiation, apoptosis, angiogenesis, and metastasis (Cargnello and Roux, 2011; Guo et al., 2020).

##### Signaling Mediator 3: Signal Transduction Pathway

In laryngeal cancer, lncRNA DGCR5 upregulated radioresistance through miR-506/Wnt pathway (Tang and Shan, 2019), and Wnt pathway is one of the key cascades that regulate caner development and stemness (Zhan et al., 2017b). Besides, in lung cancer, lncRNA AGAP2-AS1 enhanced radioresistance through miR-296/NOTCH2 axis (Zhang et al., 2021). NOTCH2 (notch receptor 2) is a member of the Notch family. The continuous Notch2 signal promotes tumor cell EMT while avoiding apoptosis, and the increase of Notch2 expression is related to the poor clinical prognosis of patients (Xiu and Liu, 2019).

#### Acting on Protein to Regulate Downstream Signals

In addition to regulating miRNAs, lncRNAs can also directly interact with specific proteins to participate in cancer radioresistance/radiosensitivity. In breast cancer, lncRNA LINC02582 directly interacted with a ubiquitinase USP7, which reduced the level of CHK1 protein, resulting in radioresistance (Wang et al., 2019). In glioma, Linc-RA1 can combine with H2B to stabilize the level of H2B K120 monoubiquitination and inhibit the interaction between H2Bub1 and ubiquitin-specific protease 44 (USP44), thus regulating autophagy and enhanced radioresistance (Zheng et al., 2020). In non-small cell lung cancer, lncRNA linc-SPRY3 could bind to IGF2BP3 (Insulin Like Growth Factor 2 MRNA Binding Protein 3), which leads to the destabilization of c-Myc and HMGA2, and improves the radiosensitivity of tumors (Brownmiller et al., 2020).

#### Acting Like Transcription Factor to Regulate Downstream Signals

Besides, lncRNAs can also act like transcription factors to initiate downstream signaling pathways and participate in tumor radioresistance/radiosensitivity. In esophageal cancer, lncRNA MAGI2-AS3 can recruit the histone methyltransferase EZH2 to the HOXB7 promoter region to initiate H3K27me3 and repress HOXB7 expression, resulting in enhanced tumor radiosensitivity (Cheng et al., 2020). In nasopharyngeal cancer, lncRNA PVT1 can act as a scaffold for the chromatin modifier KAT2A, recruiting the nuclear receptor-binding protein TIF1β to activate NF90 transcription, thereby increasing HIF-1α and upregulating radioresistance (Wang et al., 2020e). HIF-1α activates the transcription of many genes that encode proteins involved in cancer angiogenesis, glucose metabolism, cell proliferation/survival, and invasion/metastasis (Semenza, 2003). In lung adenocarcinoma, down-regulated lncRNA LINC00857 inhibited the expression of BIRC5 by inhibiting the enrichment of NF-κB1 in the promoter region of BIRC5, thereby enhancing radiosensitivity (Han et al., 2020c).

In addition, some studies have reported that lncRNAs could be involved in tumor radioresistance/sensitivity through DNA repair (Jiang et al., 2017; Li et al., 2018b; Zhang et al., 2019b; Qian et al., 2020), cell cycle regulation (Jing et al., 2015; Li et al., 2017), and EMT (Yang et al., 2016). However, the definite biological behaviors of lncRNAs remain to be further explored. Finally, we summarized the mechanism of lncRNA-induced tumor radiosensitivity/resistance according to molecular behavior of lncRNA, biological process/signaling mediator, and downstream key molecule in Table 1.

**TABLE 1:** The summary of mechanisms by which lncRNAs contribute to cancer radioresistance/radiosensitivity.

Molecular behavior of lncRNA	Biological process/signaling mediator	Downstream key molecule
Acting on miRNA by acting as a sponge to regulate downstream signals	DNA repair and chromosome stabilization	APE1, RRM2, LIG4, ATM, EEPD1, RAD18, SMC1A, YY1
	mRNA or protein stabilization	HSPA1A, STXBP4, CARM1, SMG1
	cell cycle and proliferation	CDK1, CCND1, CAMTA1, PRC1
	apoptosis-related pathway	IER3, IGF1R, E2F2, PTMA
	autophagy-related pathway	ATG5, ATG12
	epithelial-mesenchymal transition	ZEB1, ZEB2, L1CAM
	cellular energy metabolism	HK2, NR1D2
	transcription factor	FOXM1, HOXA10
	Kinase	ACK1, ErbB4, MAPK
	signal transduction pathway	Wnt, NOTCH
Acting on protein to regulate downstream signals	cell cycle and proliferation	USP7, CHK1, c-Myc
	autophagy-related pathway	H2B, USP44
Acting like transcription factor to regulate downstream signals	Induce or prevent transcription	EZH2, HOXB7, H3K27me3, KAT2A, TIF1β, BIRC5

## Discussion

Radiation therapy is one of the core methods of cancer treatment. However, cancer radiation resistance often limits the effectiveness of this treatment, and the mechanisms of radioresistance remain largely unknown. With the development of biotechnologies such as high-throughput sequencing, bioinformatics analysis, and animal modeling, lncRNAs have been shown to play critical regulatory roles in tumorigenesis and progression (Yang et al., 2014). Their role in tumor therapy resistance provided new insights for identifying appropriate treatments for specific populations, improving treatment resistance, and developing novel therapeutic targets (Zhang et al., 2020b). Therefore, exploring the detailed function of lncRNAs in tumor radioresistance/sensitivity will allow us to identify corresponding novel biomarkers and develop new lncRNA-based methods to improve radioresistance to achieve precise radiotherapy for patients. To the best of our knowledge, this study is the first of its kind to systematically evaluate the functions of lncRNAs in cancer radioresistance/sensitivity on the basis of high-quality experimental evidence.

The mechanisms by which lncRNAs participate in tumor radioresistance/sensitivity may mainly include three categories. 1) lncRNAs act on microRNA (miRNA) through a sponge, and their downstream signals include some specific molecular biological processes (DNA repair and chromosome stabilization, mRNA or protein stabilization, cell cycle and proliferation, apoptosis-related pathways, autophagy-related pathways, epithelial-mesenchymal transition (EMT), cellular energy metabolism) and some signaling mediators (transcription factors, kinases, some important signal transduction pathways) that regulate various biological processes. 2) lncRNAs directly interact with proteins to participate in tumor radioresistance through affecting the cell cycle and autophagy. 3) lncRNAs act like transcription factors to initiate downstream signaling pathways and participate in tumor radioresistance. Besides, the same lncRNA may be involved in radioresistance\sensitivity by different mechanisms in different tumors, such as lncRNA HOTAIR, lncRNA GAS5, lncRNA PVT1, lncRNA TUG1, lncRNA DGCR5, lncRNA FAM201A, lncRNA KCNQ1OT1, lncRNA LINC00958, lncRNA MALAT1, lncRNA NEAT1, and lncRNA XIST. This also revealed that lncRNAs may have multiple potential mechanisms of action in tumor radioresistance, and may act through multiple mechanisms simultaneously. Some bioinformatics methods, such as RNA-RNA binding, RNA-protein binding prediction algorithms, will provide clues to comprehensively characterize the biological behavior of lncRNAs (Rinn and Chang, 2012). Of course, further experimental verification is still the top priority.

There have been studies on the relationship between radiotherapy efficacy and lncRNAs as biomarkers for radiotherapy patients, such as in non-small cell lung cancer and glioma (Lin et al., 2020; Song et al., 2021). However, the clinical application of lncRNAs as biomarkers of radioresistance still faces huge challenges. First, in addition to collecting lncRNA data from patients who have already received radiation therapy, it is often necessary to collect lncRNA data from the control (normal) population, which is often difficult to accomplish. Organoids provide new insight into this dilemma, a method that can closely mimic the physiology of humans *in vitro* (Drost and Clevers, 2018). The expression levels of lncRNAs change dynamically, and the sample obtained represents a snapshot of the patient’s current state. However, radiotherapy is often time- and dose-dependent; therefore, it is imperative to investigate the time- and dose-dependent effects of lncRNAs on tumor radiation resistance. In addition, lncRNAs are highly tissue-specific, and it is also necessary to compare lncRNA-specific changes in different organs after irradiation. Therefore, a comprehensive understanding of the regulatory paradigm of lncRNAs in tumor radioresistance will help us to better screen verifiable, detectable, highly sensitive, and highly specific lncRNA biomarkers using novel biotechnologies.

Moreover, some lncRNA-based precision medicine clinical trials have been carried out or are underway, such as lncRNA MALAT1 (Amodio et al., 2018), lncRNA HOTAIR (Di et al., 2017). Although there is currently no study of lncRNAs used in clinical patients to improve radiotherapy resistance, studies have shown that nanoparticles-meditated LncRNA AFAP1-AS1 silencing to block the wnt/β-catenin signaling pathway can effectively improve the radioresistance of triple-negative breast cancer (Bi et al., 2020). Challenges remain until these techniques can be applied to improve tumor radiation resistance. First, due to the complex mode of function of lncRNAs, this requires further comprehensive understanding and assessment of the specific functions of lncRNAs involved in cancer radioresistance. Second, the design of the lncRNA delivery system still needs to be further optimized to improve transfection efficiency, reduce off-target effects, and prolong the half-life of lncRNA degradation. Mastering the mechanism of lncRNA in tumor radioresistance/sensitivity will help us to screen more suitable biomarkers and therapeutic targets. This systematic review provides convincing evidence for the mechanism by which lncRNAs are involved in tumor radioresistance/sensitivity. More fundamental and clinical research is needed in the future to investigate how lncRNAs affect various aspects of radioresistance/radiosensitivity, and to study the application value of lncRNAs in radiotherapy.

## Conclusion

In conclusion, this systematic review studied the mechanism of lncRNA in cancer radioresistance/radiosensitivity. The mechanisms by which lncRNAs participate in tumor radioresistance/sensitivity may mainly include three categories. 1) lncRNAs act on microRNA (miRNA) through a sponge, and their downstream signals include some specific molecular biological processes (DNA repair and chromosome stabilization, mRNA or protein stabilization, cell cycle and proliferation, apoptosis-related pathways, autophagy-related pathways, epithelial-mesenchymal transition (EMT), cellular energy metabolism) and some signaling mediators (transcription factors, kinases, some important signal transduction pathways) that regulate various biological processes. 2) lncRNAs directly interact with proteins to participate in tumor radioresistance through affecting the cell cycle and autophagy. 3) lncRNAs act like transcription factors to initiate downstream signaling pathways and participate in tumor radioresistance. Different lncRNAs may participate in the radioresistance with the same regulatory paradigm, and the same lncRNAs may also participate in the radioresistance through different mechanisms. More detailed studies on how lncRNAs are involved in tumor radioresistance are urgently needed to help us screen more suitable biomarkers and therapeutic targets. This will provide a rationale for large-scale clinical validation and may ultimately improve tumor radioresistance and patient prognosis.
