# Supplementary material for: The Mechanism of Long Non-coding RNA in Cancer Radioresistance/Radiosensitivity: A Systematic Review
Source: Front Pharmacol. 2022 May 5;13:879704. doi: 10.3389/fphar.2022.879704 (PMC9117703; doi:10.3389/fphar.2022.879704)
Supplement: Supplementary file 1 [file Table1.DOCX]

**Table S1** Search strategy based on Pubmed

| #1 | neoplasms (Mesh Terms) |
| --- | --- |
| #2 | neoplasia OR tumor OR tumour OR carcinoma OR cancer OR adenoma OR adenocarcinoma |
| #3 | #1 OR #2 |
| #4 | RNA, long noncoding (Mesh Terms) |
| #5 | lncRNA OR lincRNA OR long non-coding RNA OR long untranslated RNA OR long non-protein-coding RNA |
| #6 | #4 OR #5 |
| #7 | radiotherapy OR ionizing radiation OR ionizing OR radiation OR radioresistance OR radiosensitivity |
| #8 | #3 AND #6 AND #7 |
