# Supplementary material for: The Mechanism of Long Non-coding RNA in Cancer Radioresistance/Radiosensitivity: A Systematic Review
Source: Front Pharmacol. 2022 May 5;13:879704. doi: 10.3389/fphar.2022.879704 (PMC9117703; doi:10.3389/fphar.2022.879704)
Supplement: Supplementary file 2 [file Table2.docx]

**Table S2** Mechanisms of lncRNAs contributing to cancer radioresistance/radiosensitivity

| lncRNA | Cancer type | Radioresistance | Mechanism of regulation |
| --- | --- | --- | --- |
| lncRNA TUG1^12^ | bladder cancer | upregulate | to modulate miR-145/ZEB2 axis |
| lncRNA TUG1^13^ | bladder cancer | upregulate | to promote HMGB1 expression |
| lncRNA HOTAIR^14^ | breast cancer | upregulate | to facilitate HSPA1A expression via sequestering miR-449b-5p |
| lncRNA LINC00511^15^ | breast cancer | upregulate | to modulate miR-185/STXBP4 |
| lncRNA LINC02582^16^ | breast cancer | upregulate | to interact with USP7 to deubiquitinate and stabilize CHK1 |
| lncRNA LINC00963^17^ | breast cancer | upregulate | to modulate miR-324-3p/ACK1 axis |
| lncRNA HOTAIR^18^ | breast cancer | upregulate | to modulate EZH2 |
| lncRNA H19^19^ | cardiac cancer | upregulate | to modulate miR-130a-3p and miR-17-5p |
| lncRNA TRPM2-AS^20^ | gastric cancer | upregulate | to enhance the expression of FOXM1 by acting as a sponge of miR-612 |
| lncRNA NEAT1^21^ | gastric cancer | upregulate | to modulate miR-27b-3p |
| lncRNA LINC01436^22^ | gastric cancer | upregulate | to modulate miR-513a-5p/APE1 axis |
| lncRNA SNHG12^23^ | cervical cancer | upregulate | to modulate miR-148a/CDK1 pathway |
| lncRNA PCAT1^24^ | cervical cancer | upregulate | to modulate miR-128/GOLM1 axis |
| lncRNA LINC00958^25^ | cervical cancer | upregulate | to modulate miR-5095/RRM2 |
| lncRNA GAS5^26^ | cervical cancer | downregulate | to modulate miR-106b/IER3 axis |
| lncRNA HOTAIR^27^ | cervical cancer | upregulate | to promote HIF-1α expression |
| lncRNA NEAT1^28^ | cervical cancer | upregulate | to regulate miR-193b-3p/CCND1 axis |
| lncRNA HOTAIR^29^ | cervical cancer | upregulate | to regulate p21 expression |
| lncRNA HOTAIR^30^ | colorectal cancer | upregulate | to regulate microRNA-93/ATG12 |
| lncRNA lnc-RI^31^ | colorectal cancer | upregulate | to regulate NHEJ repair through miR-4727-5p/LIG4 |
| lncRNA LINC00958^32^ | colorectal cancer | upregulate | to modulate miR-422a/MAPK1 axis |
| lncRNA EGOT^33^ | rectal cancer | upregulate | to modulate miR-211-5p/ErbB4 axis |
| lncRNA MAGI2-AS3^34^ | esophageal cancer | downregulate | to down-regulate HOXB7 through interaction with EZH2 |
| lncRNA LINC00261^35^ | esophageal cancer | downregulate | to modulate miR-552-3p/DIRAS1 |
| lncRNA DIO3OS^36^ | esophageal squamous cell cancer | downregulate | to modulate miR-130b/PAX9 |
| lncRNA DNM3OS^37^ | esophageal squamous cell cancer | upregulate | to regulate DNA damage response |
| lncRNA FAM201A^38^ | esophageal squamous cell cancer | upregulate | to regulate ATM and mTOR expression via miR-101 |
| lncRNA NORAD^39^ | esophageal squamous cell cancer | upregulate | to modulate EEPD1/ATR/Chk1 axis and inhibit miR-199-a1 |
| lncRNA TUG1^40^ | esophageal squamous cell cancer | upregulate | to modulate miR-144-3p and MET/EGFR/AKT axis |
| lncRNA MALAT1^41^ | esophageal squamous cell cancer | upregulate | to modulate Cks1 expression |
| lncRNA HOTAIRM1^42^ | glioblastoma | upregulate | to regulate mitochondrial function and ROS levels via TGM2 |
| lncRNA HMMR-AS1^43^ | glioblastoma | upregulate | to regulate DNA repair proteins ATM, RAD51, and BMI1 |
| lncRNA RBPMS-AS1^44^ | glioblastoma | downregulate | to promote NRGN transcription through the miR-301a-3p/CAMTA1 axis |
| lncRNA TPTEP1^45^ | glioma | downregulate | to stimulate the P38 MAPK signaling through interacting with miR‑106a‑5p |
| lncRNA linc-RA1^46^ | glioma | upregulate | to prevent H2Bub1/USP44 combination |
| lncRNA TP53TG1^47^ | glioma | upregulate | to modulate miR-524-5p/RAB5A axis |
| lncRNA SNHG18^48^ | glioma | upregulate | to inhibit semaphorin 5A |
| lncRNA NCK1-AS1^49^ | glioma | upregulate | to modulate miR-22-3p/IGF1R |
| lncRNA lincRNA-p21^50^ | glioma | upregulate | to regulate β-catenin |
| lncRNA LINC01123^51^ | glioma | upregulate | to modulate miR-151a/CENPB axis |
| lncRNA XIST^52^ | glioma | upregulate | to modulate miR-329-3p/CREB1 axis |
| lncRNA LINC00520^53^ | head and neck squamous cell cancer | upregulate | to modulate miR-195/HOXA10 |
| lncRNA HOTAIR^54^ | laryngeal cancer | upregulate | to modulate miR-454-3p/E2F2 axis |
| lncRNA DGCR5^55^ | laryngeal cancer | upregulate | to modulate miR-506/Wnt pathway |
| lncRNA DGCR5^56^ | laryngeal cancer | upregulate | to regulate miR-195 |
| lncRNA NEAT1^57^ | nasopharyngeal cancer | downregulate | to modulate miR-101-3p/EMP2 axis |
| lncRNA PVT1^58^ | nasopharyngeal cancer | upregulate | to stabilize HIF-1α through promoting the binding between H3K9ac and TIF1β |
| lncRNA linc00312^59^ | nasopharyngeal cancer | downregulate | to target DNA-PKcs and impairing DNA damage repair |
| lncRNA MINCR^60^ | nasopharyngeal cancer | upregulate | to modulate miR-223/ZEB1 |
| lncRNA LINC00114^61^ | nasopharyngeal cancer | upregulate | to regulate ERK/JNK signaling pathway via targeting miR-203 |
| lncRNA PVT1^62^ | nasopharyngeal cancer | upregulate | to modulate miR-515-5p/PIK3CA |
| lncRNA CASC19^63^ | nasopharyngeal cancer | upregulate | to promote autophagy via AMPK-mTOR pathway |
| lncRNA ANCR^64^ | nasopharyngeal cancer | upregulate | to inhibit PTEN expression |
| lncRNA LINC-PINT^65^ | nasopharyngeal cancer | downregulate | to inhibit DNA damage repair through ATM/ATR-Chk1/Chk2 |
| lncRNA PTPRG-AS1^66^ | nasopharyngeal cancer | upregulate | to modulate miR-194-3p/PRC1 |
| lncRNA MALAT1^67^ | nasopharyngeal cancer | upregulate | to modulate miR-1/slug axis |
| lncRNA KCNQ1OT1^68^ | hepatocellular cancer | upregulate | to modulate miR-146a-5p/ACER3 axis |
| lncRNA TP73-AS1^69^ | hepatocellular cancer | upregulate | to modulate PTEN/Akt pathway |
| lncRNA ROR^70^ | hepatocellular cancer | upregulate | to modulate miR-145/RAD18 axis |
| lncRNA GAS5^71^ | hepatocellular cancer | downregulate | to modulate miR-144-5p/ATF2 axis |
| lncRNA MIR22HG^72^ | hepatocellular cancer | downregulate | to promote the production of miR-22-5p |
| lncRNA LINC00483^73^ | lung cancer | upregulate | to modulate miR-144/HOXA10 |
| lncRNA HOTAIR^74^ | lung cancer | upregulate | to regulate β-catenin |
| lncRNA AGAP2-AS1^75^ | lung cancer | upregulate | to modulate miR-296/NOTCH2 |
| lncRNA LINC00461^76^ | lung cancer | upregulate | to modulate miR-195/HOXA10 |
| lncRNA LINC00857^77^ | lung adenocarcinoma | upregulate | to form functional regulatory LINC00857-NF-κB1-BIRC5 triplet |
| lncRNA KCNQ1OT1^78^ | lung adenocarcinoma | upregulate | to induce ATG5/ATG12-mediated autophagy via miR-372-3p |
| lncRNA SBF2-AS1^79^ | non-small cell lung cancer | upregulate | to modulate microRNA-302a/MBNL3 axis |
| lncRNA FAM201A^80^ | non-small cell lung cancer | upregulate | to upregulate EGFR and HIF-1α via miR-370 |
| lncRNA PVT1^81^ | non-small cell lung cancer | upregulate | to regulate miR-195 |
| lncRNA PVT1^82^ | non-small cell lung cancer | upregulate | to modulate miR-424-5p/CARM1 |
| lncRNA CYTOR^83^ | non-small cell lung cancer | upregulate | to modulate miR-206/PTMA axis |
| lncRNA CBR3-AS1^84^ | non-small cell lung cancer | upregulate | to modulate miR-409-3p/SOD1 |
| lncRNA HNF1A-AS1^85^ | non-small cell lung cancer | upregulate | To modulate miR-92a-3p/MAP2K4/JNK axis |
| lncRNA linc-SPRY3^86^ | non-small cell lung cancer | downregulate | to interact with IGF2BP3 and affect RNA stability in HMGA2 and c-MYC mRNAs |
| lncRNA GAS5^87^ | non-small cell lung cancer | downregulate | to modulate miR-135b |
| lncRNA RBM5-AS1^88^ | medulloblastoma | upregulate | to stabilization of SIRT6 protein |
| lncRNA LINC00518^89^ | melanoma | upregulate | to regulate glycolysis through an miR-33a-3p/HIF-1α negative feedback loop |
| lncRNA LINC01224^90^ | melanoma | upregulate | to modulate miR-193a-5p/NR1D2 axis |
| lncRNA XIST^91^ | neuroblastoma | upregulate | to modulate the miR-375/L1CAM |
| lncRNA LINC01410^92^ | neuroblastoma | upregulate | to modulate miR-545-3p/HK2 axis |
| lncRNA HULC^93^ | prostate cancer | upregulate | to modulate autophagy via Beclin-1 and mTOR |
| lncRNA TUG1^94^ | prostate cancer | upregulate | to modulate miR-139-5p/SMC1A axis |
| lncRNA GAS5^95^ | prostate cancer | downregulate | to modulate miR-320a/RAB21 axis |
| lncRNA LINC02532^96^ | renal cell cancer | upregulate | to modulate miR-654-5p/YY1 axis |
| lncRNA SNHG7^97^ | thyroid cancer | upregulate | to modulate miR-9-5p/DPP4 axis |
| lncRNA GAS5^98^ | thyroid cancer | downregulate | to modulate miR-362-5p/SMG1 axis |
